# How to improve access to health care for Roma living in social exclusion: a concept mapping study

**DOI:** 10.1186/s12939-021-01396-4

**Published:** 2021-02-12

**Authors:** Ivana Svobodova, Daniela Filakovska Bobakova, Lucia Bosakova, Zuzana Dankulincova Veselska

**Affiliations:** 1 Palacky University in Olomouc, Olomouc University Social Health Institute, Olomouc, Czech Republic; 2 Department of Health Psychology and Research Methodology, University of Pavol Jozef Safarik in Kosice, Faculty of Medicine, Kosice, Slovak Republic

**Keywords:** Health care access, Vulnerable population, Roma, Ethnicity, Policies, Interventions, Concept mapping, Czech Republic

## Abstract

**Background:**

Half of the people living in social exclusion in the Czech Republic are of Roma origin. The worse health of Roma could be partly explained by numerous barriers to accessing health care. Therefore, our study aimed to explore the perceptions of various stakeholders and experts who may have an impact on the inclusion of Roma and/or their access to health care on how to improve health care access for Roma living in social exclusion in the Czech Republic.

**Methods:**

We conducted a concept mapping study and obtained data from 32 participants from health and social services, policymakers and others who were involved in different study phases (brainstorming, sorting, rating, interpretation).

**Results:**

Out of 64 proposed measures sorted into six distinct clusters, 20 were rated as the most urgent and the most feasible and should be implemented with a priority to improve access to health care for Roma living in social exclusion. The proposed measures covered various topics, such as education and awareness of the target group as well as education and supervision of helping professionals, strengthening capacities and streamlining the health care system, health promotion and associated services and increasing the local and financial accessibility of health care. Overall, measures concerning the education and supervision of helping professionals were rated as both the most urgent and the most feasible. Individual priority measures targeted, for example, the health needs assessment of Roma living in social exclusion to set up interventions or to include topics such as participation, empowerment, cultural competence and communication training in the curricula of health care and helping professionals in postgraduate and continuing studies.

**Conclusions:**

Stakeholders proposed a set of relevant and acceptable measures that may help improve access to health care for Roma living in social exclusion. The way they rated the proposed measures reflects both the current unfavourable mainstream and public discourse concerning Roma living in social exclusion and the most acute policy issues identified by several European and national bodies.

**Supplementary Information:**

The online version contains supplementary material available at 10.1186/s12939-021-01396-4.

## Introduction 

Health systems play an important role in mediating the differential consequences of illness in people’s lives [1]. Health care is estimated to account for 10–20% of the malleable contributors to healthy outcomes for a population [2]. Its accessibility is an important factor of the social determinants of health [3], bringing disparities in health outcomes when not equally accessible [4]. Addressing the social determinants of health [3], including health care accessibility, is significant for reducing longstanding health disparities. According to the WHO, universal access to health care is a priority that could be guaranteed and with acceleration extended to all countries in the EU region [5]. Accessibility is integrated into the concept of Universal Health Coverage, which implies that all people receive all the health care they need (preventive care, providing treatment, rehabilitation and palliative care) of sufficient quality and that the use of the particular services does not lead to financial hardship [6].

There are several points of view on how to define barriers in access to health care. Gelberg-Andersen’s behavioural model shows the contextual and individual characteristics of the population (predisposing, enabling, and need characteristics) that affect people’s use of health services [7]. Pechansky and Thomas proposed a taxonomic definition of “access”, which summarizes a set of five specific dimensions [8]. Levesque et al. defined five similar dimensions of accessibility of services and five corresponding abilities of persons: 1) Approachability – Ability to perceive; 2) Acceptability – Ability to seek; 3) Availability and accommodation – Ability to reach; 4) Affordability – Ability to pay; 5) Appropriateness – Ability to engage [9]. For our research, we define access to health care according to Levesque as the “opportunity to reach and obtain appropriate health care services in situations of perceived need for care” [9].

The Roma are the largest ethnic minority in Europe [10] and also in the Czech Republic. The latest, most realistic estimate is that they number about 240,300 (2.2%) from the whole population, according to the Office of the Government in a report from 2018 [11]. Many Roma have been caught in a cycle of social exclusion and financial hardship for generations [12]. Since the sixteenth century, the history of Roma in the Czech Republic has been marked by intolerance, punitive policies, and assimilation. Both communist regime policies and the transition to democracy and capitalism after 1989 widened the social and economic gap between Roma and the majority population [13]. The lower education and higher unemployment rates [14], underlined by constant racism, oppression and marginalisation [15], resulted not only in segregation but also in self-exclusion [16]. At present, about 50% of Roma in the Czech Republic live in social exclusion [17]. In Central and Eastern Europe, the health status of Roma has been reported as persistently poorer than that of the general population [18, 19], which applies for the Czech Republic as well [20]. Roma have an earlier incidence of chronic illnesses and higher untimely mortality (by about 10–15 year on average) as well as other diseases [21]. Roma ethnicity has been found to be provably associated with worse self-rated health status in general [22–24] and also in vulnerable groups of the Roma population: infants [25], children [26] and women [27].

The worse health of Roma could be partially explained by higher vulnerability [28] but also by numerous barriers in accessing health care [29–31], which are significantly higher than for the non-Roma population [29, 30]. All of these barriers are aggravated by an absence of personal identification documents, distance or the discriminatory approach of health professionals [30]. Research has shown barriers in both — health professionals and Roma —which are structural, economic, cognitive or psychological in nature [28, 31]. Improving access to health care for vulnerable populations is one of the aims of the European Union [32] and also the WHO [33].

Prevailing strategies based on a top-down approach have not effectively regulated the problems of vulnerable Roma groups [34]. Participative research is the co-construction of research among researchers, people who are concerned with the subject of the study (e.g. patients, community members, health professionals, and representatives of organizations) and/or decision-makers who can create sustainable system modifications, and it is relevant for a broad spectrum of health research [35]. Such a partnership is a crucial part of this type of research; it brings to the centre of knowledge production key actors who have the potential to offer and apply practical solutions that are more appropriate to their specific needs and supports the use of the knowledge acquired from research for specific activities [36]. Participative research in the area of supporting health improves research quality, empowerment and the sustainability of the results [35] contributes to the consolidation of the needed tools or changes to the national health policy [37]; and allows rapid implementation of the study results back into health care practise or the community [38]. Applied participative research is for these reasons recommended for “Roma health” research, because it can bring processes that may have a higher potential to increase the health and prosperity of Roma [39]. Concept mapping is a participative method used for over 20 years in many fields of research, including health care, that brings strong interior representational validity and reliability [40].

There have been several attempts to explore the consequences connected to barriers to health care, which were rather partial: health insurance [41] and disparities in having unmet health needs [42, 43]. We, however, found only a few studies using a participatory approach, one of which was focused on health literacy and empowerment [44] and another on Roma health justice [34]. We further found only one concept mapping study intended to improve access to primary health care for Roma women experiencing intimate partner violence [45].

All the studies mentioned above addressed only partial issues connected to the complex topic of health care access for the vulnerable population of Roma living in social exclusion, with a participatory approach scarcely used. Moreover, we did not find any participatory study focused on how to improve access to the health care for this vulnerable group. Therefore, our study aimed to explore how to improve access to health care for Roma living in social exclusion in the Czech Republic and on priority measures, based on their urgency and feasibility, using the concept mapping approach.

## Methods

This study was carried out within the TA CR-funded ROMZAVIP Project (TL02000164), which aimed to innovate the existing methodologies and procedures concerning the integration of Roma living in social exclusion in the area of health care accessibility. Within this project, the triangulation of different research methods and data was used to increase the validity of the findings [46]. First, all key actors and stakeholders involved in the topic were mapped and qualitative analysis of documents related to social inclusion and health was performed. Next, qualitative research focused on the social determinants of health with an emphasis on the access to health care from the perspective of all stakeholders, including Roma living in social exclusion, was conducted. This was followed by a multiple case study that focused on the experiences and perspectives of Roma living in social exclusion regarding access to health care and the role of Roma health mediators. Next, a concept mapping (CM) study with professionals involved in health care and/or social inclusion (not only) in the area of health was conducted. Finally, the results from the CM were discussed in focus groups with Roma living in social exclusion. This project was conducted in the north-eastern part of the Czech Republic, which is characterized by a high concentration of municipalities with socially excluded locations and is among the regions most affected by social exclusion [17]. The following sections describe only the methodology and results of the concept mapping study, which is the subject of this article.

### Design

We conducted a concept mapping (CM) study with professionals involved in health care and/or social inclusion (not only) in the area of health. The CM methodology enabled us to structure perceptions of various stakeholders and experts from different fields who may have an impact on the researched topic [47]. Different working fields of stakeholders bring different perspectives and narratives for understanding the topic of the research [48], which could be inspiring for those who are in direct contact with the target group [49].

CM is a method for assessing how study participants cluster their conceptual assessment of a particular topic by developing a conceptual framework with a visual display of the clustering [47]. Using this method allowed us to use a participatory approach, with stakeholders’ involvement and visualisation of results in a way accessible and understandable for various groups [50].

### Sample

We recruited participants following Kane and Trochim [47] to ensure the availability of a wide variety of viewpoints and to support a broader range of people to adopt the resulting conceptual framework. The involvement of diverse stakeholders within all phases of our study was important to ensure broader support [51]. We involved a variety of stakeholders engaged in the studied topic or responsible for some parts of it. We used purposive sampling techniques to recruit participants from helping professionals at different levels of the work placement/hierarchy from the following fields: social services (also specialized to help with access to the health care), health care, educational institutions and authorities at the local and regional level. Part of this sample was made up of Roma who come from or reside in socially excluded areas; most of them work there daily and know the specifics and needs of the local target group very well. Besides professionals, we also aimed to include stakeholders competent to enforce the suggested measures on the policy level (e.g. directors or managers of different national institutions and local authorities).

The initial sample for brainstorming consisted of 25 participants recruited from the research region. The sorting-rating sample consisted of the same 25 experts who participated in the brainstorming session together with 7 new participants (32 altogether). This is fully following the CM methodology, as some changes in the number of participants between the brainstorming and sorting-rating step are counted, and the sample size for each step was sufficient to meet the statistical requirements to bring valid and reliable results [52]. The number of participants and their distribution according to sex, field of expertise and parts of the research that they participated in are shown in Table 1.

**Table 1:** Participants´ characteristics (total of 32 participants for sorting and rating phase)

	Number	%
Gender		
Male	11	34
Female	21	66
Field of work		
Social services	18	56
Health care	6	19
Educational institutions	5	16
Other^1^	3	9
Age		
18–25 years	1	3
26–35 years	10	31
36–45 years	10	31
46–55 years	7	22
56 years or older	2	6
did not state	2	6
Education level		
Primary	1	3
Secondary	3	9
Higher	28	88
Contact with the aimed group at work		
direct	19	59
indirect	13	41
The extent of work that directly concerns the target group		
0–25%	8	25
26–50%	4	12.5
51–75%	4	12.5
76–100%	16	50

^1^ mostly local authorities

### Procedure

The procedure consisted of five steps, three of which were related to data collection: (1) preparation, (2) brainstorming, (3) sorting and rating, (4) analysis and (5) interpretation, as suggested by Schröter et al. [51] and Kane and Trochim [47].

In the first *preparation* step conducted in December 2019 and January 2020, we identified the focal question (the core question), selected potential participants and determined the time and logistics schedule. This also included a pilot CM brainstorming with a small group of stakeholders and academics/students in which we discussed and tested the focal question. The final focal question was set as follows: “What should be done to improve the accessibility of the health care for Roma living in social exclusion?”

In the second step, conducted in February 2020, we organized a *brainstorming* session. To avoid pressure between different levels of the work placement and hierarchy, participants worked in 5 independent working groups: health professionals (physicians, nurses); officials and managers (from municipalities, local authorities, various organizations, agencies and non-governmental organizations - NGOs); health and social services personnel specialized in access to health care (from two regional agencies); social workers and educational professionals (from NGOs and primary schools) and online participants (mostly physicians who were not able to participate due to work duties). Each working group was led by a trained and experienced facilitator. The focal question was introduced and participants were encouraged to brainstorm as many ideas and measures answering the focal question as possible [47]. We obtained 129 measures. The research team reduced the duplicities, removed or merged overlapping ideas and excluded suggestions or measures that were out of the target range or not directly answering the focal question. The list of measures was further discussed with participants to refine the formulation of suggested measures as accurately and specifically as possible and to reach final agreement on the content of the final list of measures – a Master list [47]. The final Master list contained 64 measures.

The third step was *sorting and rating*, which was conducted in May 2020 via the groupwisdom™ software. Participants were asked to categorize 64 proposed measures into logically interconnected groups of similarly themed measures and to create a descriptive label for each of them. Next, participants rated measures according to two selected domains of interest: urgency (Likert scale: 1—not urgent at all, 4—very urgent) and feasibility (Likert scale: 1—very hardly feasible, 4—very easily feasible). A short, descriptive demonstration and personal assistance of the research team was available for participants to guide them during the sorting and rating process.

The fourth step was *analytical*. Before statistical analyses, a quality review of the data obtained in sorting and rating was performed to exclude those participants who did not follow the sorting and/or rating guidelines, did not complete at least 75% of the task or who provided negligent answers. On these bases, from a total of 32 participants for sorting and rating, 4 participants were excluded from the analyses of sorting and 0 from the rating. The final sample thus consisted of 28 participants for sorting analyses and 32 participants for rating analyses. All data were analysed using the groupwisdom™ software. Sorting data were analysed using multidimensional scaling to generate a point map, where the measures were plotted based on the number of times participants grouped them together, with those that were frequently grouped together positioned close to each other [45]. Hierarchical cluster analysis was conducted to generate cluster maps, where the measures were aggregated into clusters based on their proximity to each other in the point map [45]. The findings of this analysis were discussed with the advisory board consisting of the research team and external experts, following the CM methodology [47]. This advisory group chose a varying maximum number of clusters (5–11, i.e. the highest and the lowest desired number of clusters, based on the participants sorting data) and discussed the final cluster solution. The advisory board proposed 6 cluster solutions, which was also most frequently used by the participants during the sorting phase (modus).

Finally, the outcomes of the analyses (a 6-cluster solution cluster point map, rating maps and Go-Zone map) were discussed within the *interpretation* workshop held in June 2020. The interpretation group consisted of selected stakeholders who participated in the previous steps of the CM (3 health professionals, 8 social workers/managers, 3 officials). During this workshop, the interpretation group participants were asked to review the groups of measures and to discuss the proposed names for them and to adjust the labels of the groups to their final form. Then participants also discussed the set of priority measures from the Go-Zone (of a total of 64 measures), which were rated as the most urgent and most feasible by the participants in the previous rating step.

## Results

### How did participants cluster the measures related to improved access to health care for Roma living in social exclusion? - final cluster solution (cluster point map)

The final six-cluster solution was chosen and approved by the interpretation group. The interpretation group also agreed on the final labels of clusters as follows:

- Cluster 1 represents Streamlining the health care system and associated services to legitimize and take into account the needs of the target group.
- Cluster 2 represents The role of health-promotion services and access to health care.
- Cluster 3 represents Education and awareness of the target group
- Cluster 4 represents Increasing the local and financial accessibility of health care
- Cluster 5 represents Strengthening the networks and capacities of health care and prevention
- Cluster 6 represents Education and supervision of helping professions

**Fig. 1:**
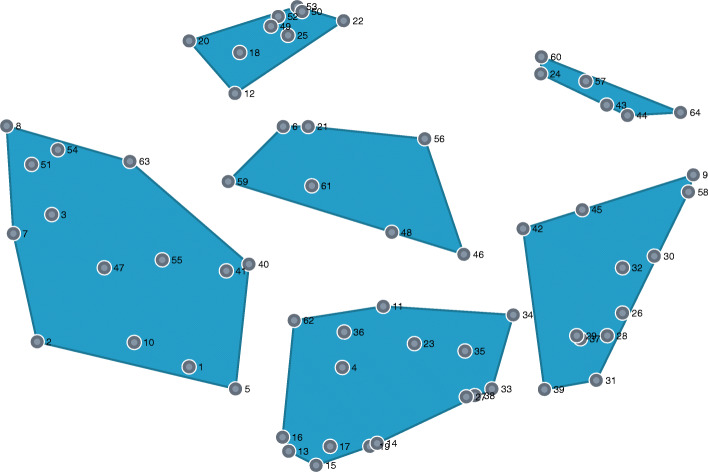
Cluster point map – final 6 clusters solution, Czech Republic, 2020. Note: Each point represents a single measure suggested by participants. The distance between the points indicates how often participants sorted particular measures into the same group (lower distance means that more participants placed particular measures into the same pile/group). The size of the cluster indicates how related these various measures are

All proposed measures and clusters can be seen in [Additional file 1]. The cluster point map is shown in Fig. 1. In this map, a point (dot) represents a measure, and the distance between the points indicates the likelihood that participants have placed the measures concerned in the same group; the clusters represent discrete groupings of related measures. The stress index was 0.2740, suggesting a strong fit between the cluster map and the data. Each point on the cluster map represents one specific measure suggested by participants.

### Which clusters mattered more in terms of urgency and feasibility according to the participants? The rating of clusters by urgency and feasibility (cluster rating maps)

**Fig. 2:**
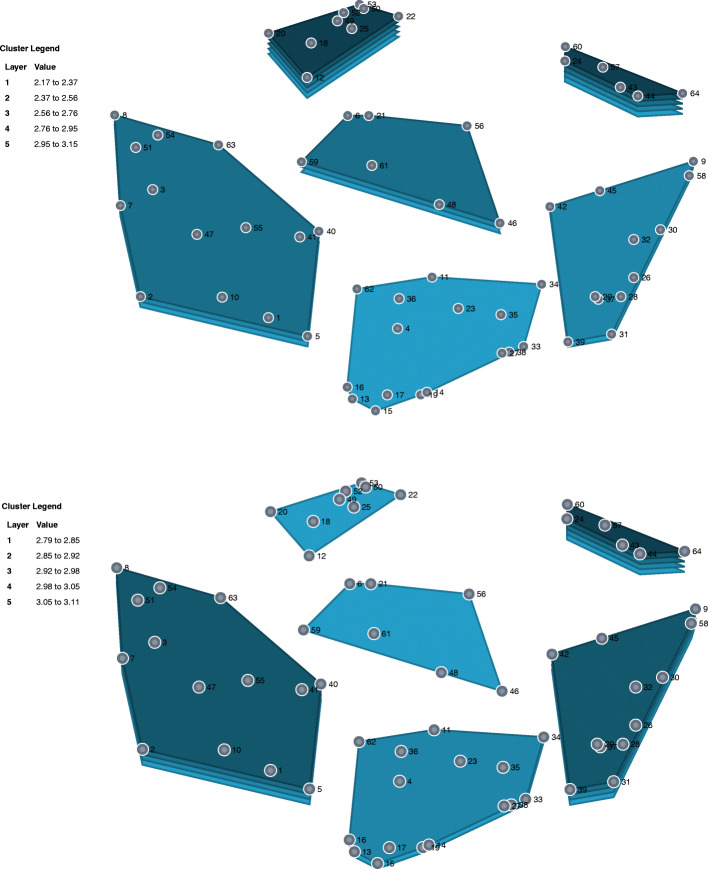
Urgency and feasibility of measures per cluster: Cluster rating maps, Czech Republic, 2020. Note: more layers indicate more urgency and feasibility

The urgency and feasibility of the various clusters as rated by participants are shown in Cluster rating maps (Fig. 2), where a third dimension (layer) displayed on top of the clusters represents the mean ratings of the selected criteria (urgency; feasibility) across all items, while the number of layers represents the higher or lower mean ratings related to other clusters in the map.

As regards urgency, participants considered Cluster 6, related to the education and supervision of helping professions, as the most urgent. Cluster 3, related to the education of the target group, and Cluster 2, related to the role of health promotion services, were rated by the participants as the least urgent.

In terms of feasibility, Cluster 3, related to the education of the target group, and Cluster 6, related to the education and supervision of the helping professions, were rated by participants as the most feasible. On the other hand, Cluster 4, related to increasing the local and financial accessibility of health care, was rated by the participants as the least feasible.

In terms of urgency and feasibility, a match occurred in Cluster 6, which was seen to be both very urgent and highly feasible. We found the biggest difference in Cluster 3, which was seen by participants as the most feasible but the least urgent, and Cluster 5, seen as very urgent but less feasible. Cluster 4, on the other hand, was rated by the participants as the least urgent and the least feasible. Table 2 shows the ranges of urgency and feasibility per cluster.

**Table 2:** Urgency and feasibility of measures per cluster: mean scores and ranges, Czech Republic, 2020

	Cluster (group)	1	2	3	4	5	6
Number of measures		14	7	9	16	12	6
Urgency	Mean (SD)	3.00 (0.21)	2.83 (0.28)	2.79 (0.32)	2.91 (0.29)	3.00 (0.26)	3.11 (0.16)
	Range (min-max)	2.53–3.26	2.19–3.13	2.31–3.19	2.38–3.31	2.45–3.34	2.94–3.35
Feasibility	Mean (SD)	2.60 (0.32)	2.66 (0.21)	3.15 (0.22)	2.17 (0.25)	2.38 (0.31)	3.00 (0.19)
	Range (min-max)	2.09–3.25	2.34–2.97	2.68–3.47	1.75–2.75	1.97–3.03	2.75–3.34

SD Standard deviation; ^a^ Mean values per clusters—higher scores indicate more urgency and more feasibility; ^b^ Minimum and maximum values of measures per clusters; the possible range of values was 1–4

### Which measures were seen as a priority according to participants? The rating of individual measures by urgency and feasibility (go-zone map)

**Fig. 3:**
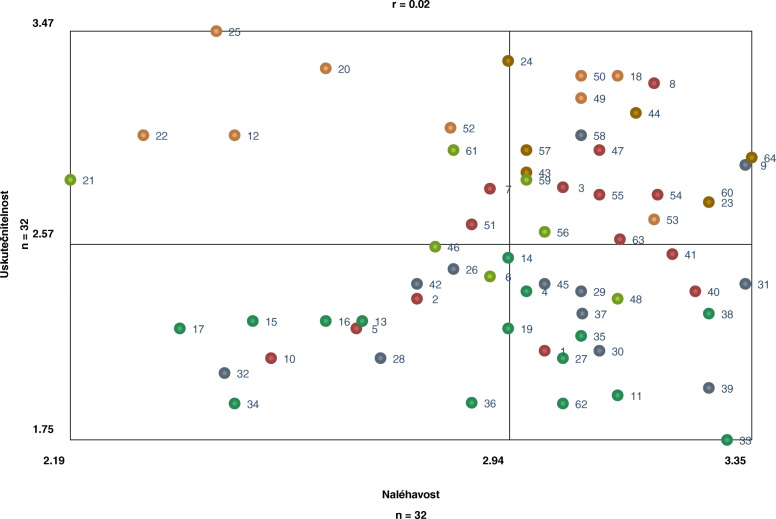
Ratings of individual measures by urgency and feasibility: Go-Zone, Czech Republic, 2020. Note: Each dot represents each measure. The X-axis shows the range of mean values for urgency (2.19–3.35); the Y-axis shows the range of mean values for feasibility (1.75–3.47). Dots in the green upper-right quadrant indicate measures that were rated above the mean (most urgent and feasible)

In the Go-Zone map (Fig. 3) the priority measures rated as the most urgent and the most feasible are placed in the green sector in the upper-right corner. Out of 64 proposed measures, 20 were rated as the most urgent and most feasible and should be, according to the participants, implemented with a priority to improve access to health care for Roma living in social exclusion. Overall, participants considered measure 8 (to explore the field of health needs of Roma living in social exclusion: what they need and expect from health care to set up interventions in the helping professions) to be the most urgent and feasible. In contrast, the least urgent and feasible, according to the participants, was measure 34 (to make the study of medicine conditional on the completion of a certain number of years in the Czech Republic or the payment of study costs, so that doctors do not go abroad (or at least in cases where a scholarship has been provided)).

Priority measures belong to each of the six clusters. The highest number of priority measures belong to Cluster 1 (*Streamlining the health care system and associated services to legitimize and take into account the needs of target groups*), whereas only one of the priority measures belongs to Cluster 4 *(Increasing the local and financial accessibility of health care*). A list of all (20) priority measures divided by clusters can be found in ***Table*** ***3***.

**Table 3:** All individual measures that were rated as the most urgent and feasible divided by clusters: Go-zone, Czech Republic, 2020

Cluster number	Measure number	Focal question: “What should be done to improve the accessibility of health care for Roma living in social exclusion?”	Urgency Scale [2.9688]–[3.3548] Median = 3.1904 *n* = 32	Feasibility Scale [2.5938]–[3.2813] Median = 2.8907 n = 32
		R = 0.021504217104074	Average Rating	Average Rating
1	47	To clearly define the competencies and scope of organizations focusing on health promotion and health care access for the purpose of regional balance of the network of helping organizations (networking, coordination of projects)	3.09	2.97
1	55	To strengthen the level of health care and prevention in socially excluded localities through the use and training of volunteers from the community, with the subsequent opportunity to later work as a “health mediator/health assistant”	3.09	2.78
1	3	To create a platform of organizations that would advocate health support for people living in social exclusion, including Roma, and try to influence public policy in this regard	3.03	2.81
1	8	To explore the field of health needs of Roma living in social exclusion: what they need and expect from health care to set up interventions in the helping professions	3.19	3.25
1	54	**To ensure the connection of clients from the target population with health care providers (strengthening their trust, lowering barriers) through health assistants and health mediators**	**3.19**	**2.78**
1	63	**To link health care with the social services available in a region (inform health care professionals about the offer and competencies of social services in the given locality, so that they are capable of turning to social services as needed and invite them to cooperate with patients – e.g. a medical counsellor for doctors, a social service mobile app)**	**3.13**	**2.59**
2	56	**Support for the sustainability and expansion of the network of “health mediators/health assistants” to all regions (fieldwork in the area of health promotion and health care access) ideally from among members of socially excluded communities with appropriate accredited training**	**3.00**	**2.63**
2	59	**Strengthening the health promotion agenda, including the availability of health care, in social prevention services (in the work of social workers and social services workers) with appropriate accredited training**	**2.97**	**2.84**
3	18	To inform clients being listed and delisted from Labour Office records about the contexts of health and social insurance and to provide them support with a risk-free resolution of a situation	3.13	3.28
3	49	Education of clients regarding medicines without a co-payment and alternatives when picking up medicine	3.06	3.19
3	50	Education of clients on the change/loss of insurance and their obligations in the area of health care provision	3.06	3.28
3	53	To ensure increased health literacy and motivation of the target population regarding health care and adequate use of health care	3.19	2.68
4	23	To introduce regular preventive paediatric and dental care in nursery schools and primary schools	3.28	2.75
5	9	To engage medical students as field health professionals within their compulsory internship	3.34	2.91
5	58	To involve students of helping professions at universities (e.g. medical disciplines, social work, medical social care, etc.) in preventive health promotion programmes, including health care accessibility	3.06	3.03
6	43	To ensure accessible supervision in the form of lifelong learning for health professionals providing care to the target population and for “health mediators/health assistants”	2.97	2.88
6	44	Education of midwives so that they can work with new mothers in obstetrics wards; education of women after childbirth in a maternity hospital (care of the self and the new-born child)	3.16	3.13
6	57	To ensure a quality workload of training for “health mediators/ health assistants” (fieldwork in the area of health promotion, including health care access) under the sponsorship of a professional guarantor based on good practices in our country and abroad (involve organizations that are already working on it) and to ensure the possibility of exchange stays	2.97	2.97
6	60	To motivate Roma children to study health disciplines and to educate and support Roma health professionals (doctors, nurses, public health protection and support assistants, midwives, paramedics, orderlies) – subsidies for scholarship and mentoring programmes (securing a network of mentors with credit motivation for students)	3.28	2.75
6	64	To educate health care and helping professionals in postgraduate studies and continuing education (equal access and ethics, participatory and supportive – empowering approaches at work, the specifics of culturally different groups of patients and related training in communication skills of health professionals related to them)	3.35	2.94

## Discussion

The aim of this study was to explore how to improve access to health care for Roma living in social exclusion in the Czech Republic using the concept mapping approach involving stakeholders from various fields of policy and practice. Participants proposed 64 measures sorted in 6 distinct clusters, with Cluster 6, representing the education and supervision of helping professions, being the most urgent and most feasible, while Cluster 4, on increasing the local and financial accessibility of health care, the opposite. The biggest difference was found in Cluster 3 (education of the target group), which was seen by participants as the most feasible but least urgent, and Cluster 5 (strengthening the capacities of health care), seen as very urgent but less feasible. Overall, 20 individual measures were rated as the most urgent and feasible and should be implemented with the priority to improve access to health care for Roma living in social exclusion. The proposed measures covered a variety of topics, such as education and awareness of the target group, education and supervision of helping professions, strengthening capacities and streamlining the health care system, health promotion and associated services, and increasing the local and financial accessibility of health care. Both upstream as well as downstream interventions and policy measures were introduced by the participants. Several measures contain a participatory approach and count on the participation of the target group. Overall, the proposed measures are in line with the European Pillar of Social Rights [53], the recommendations of an EU expert panel [32] and the WHO [54, 55] concerning the improvement of access to healthcare for vulnerable groups (including Roma), making them acceptable and appropriate for implementation at the local and most of them at the national level.

We found that Cluster 6, related to education and supervision of helping professions, was rated as the most urgent and feasible. This cluster contains measures targeting quality and accessibility of lifelong education and supervision for various professions, such as health care professionals, midwives, social workers, health mediators and others. The relevance of the individual proposed measures included in Cluster 6 is supported by recommendations from several other policy papers and strategies [56–59]. One of the suggested measures (No. 60) aims to motivate Roma children to study health disciplines, which corresponds with the newest evidence-based policy recommendations on how to combat structural anti-Gypsyism [56, 60]. The suggested measures might be put into the practice by educational or non-governmental institutions. It would be appropriate to include these goals into strategic or action plans under the responsibility of the relevant ministries (Ministry of Health, Education and Social Affairs) or to applicable laws and related regulations.

We further found two clusters which were rated as contradictory in terms of urgency and feasibility. The first was Cluster 3, related to education and awareness of the target group, which was rated as the least urgent but the most feasible. We might speculate that the logic behind such a rating is that these activities towards people at risk of social exclusion are in progress already; thus, the stakeholders view them as very feasible and not urgent. Many of the proposed measures included in this cluster are part of European and national strategies [61] and have already been implemented by different governmental and non-governmental organizations, for example by National Health Institute [20] and NGOs providing community social services, such as DROM, which specializes in and focuses directly on social health support [62]. However, the recent study of Rolantova, Kajanova and Manhalova shows that health literacy among Roma in the Czech Republic remains low [63]. This may be due to the lack of action on other determinants of health causing health promotion activities to be less efficient. Education can be a good choice only as part of complex systemic changes [64]. Responsibility for the implications of the proposed measures can remain at current organizations, but according to our results, more attention should be given to structural changes that can promote health equity [65].

The second contradictory-rated cluster was Cluster 5, related to strengthening the networks and capacities of health care and prevention, which was seen by the participants as very urgent but less feasible. The shortage of primary care doctors and regional disparities in the Czech Republic are among the key challenges [66], and capital investments are too low to sustain effective infrastructure [66]. This directly corresponds with the rating of this cluster, because the solution is long-term and rests on structural changes. The primary care reform that can solve this issue is a part of the new national strategy, Health 2030, that is in the authority of the Ministry of Health [67].

Cluster 4, related to increasing the local and financial accessibility of health care for the target group, was rated as less urgent and the least feasible. Most of the measures in this cluster concern reducing costs for citizens with low income. Worse accessibility of health care for households with low income is a long-term issue in many EU countries, including the Czech Republic [68]. It is assumed that effective solutions of financial accessibility will put an excessive strain on the health care system budget [68], which may be the reason why participants see this cluster as less feasible. Moreover, participants rated this cluster as less urgent despite the fact that the target group concerns people of low income and therefore the financial barrier is significant. Overall, such a rating most likely reflects the negative stereotypes that seem to be still present in mainstream and public discourse about “Roma” in the Czech Republic [58]. A common belief is that Roma are consciously abusing the social system [69], so this target group is probably considered to be less deserving than elderly, sick or disabled people [70]. Increasing benefits, such as those related to health care, for a group perceived to be unemployed often causes tensions in society [71], which is why a high level of conditionality is applied regarding access to social benefits in European states [72]. Implementation of potentially unpopular measures into social policies may be carried out by the government in cooperation with the Ministry of Health and followed by an evaluation of their financial and social impact.

Participants identified 20 priority measures which they evaluated as the most urgent and feasible (see Table 3 for all of them). More than half of these priority measures were from Clusters 1 and 6. Six priority measures from Cluster 1 (Streamlining the health care system and associated services to legitimize and take into account the needs of the target group) place emphasis on the role, competencies, capacities and cooperation of institutions and organizations involved in advocacy, social services, health promotion and health mediation. Their active involvement in the target group and cooperation with health care providers seems to be crucial in helping Roma living in social exclusion to overcome their specific barriers in access to health care [28, 29]. Five priority measures from Cluster 6 (Education and supervision of helping professions) show that there is also urgent need to overcome existing barriers on the side of helping professionals and increase the quality of services they provide through education, training and supervision [28, 60]. Another four priority measures, from Cluster 3 (Education and awareness of the target group), are focused on health literacy in general but also more specifically related to rights and obligations regarding health and social insurance and unnecessary co-payments for medicines. The rest of the priority measures target strengthening capacities of health mediation and health promotion in particular through the involvement of students of relevant disciplines as well as community members.

In the following paragraph, the top three highest rated individual measures will be discussed. The highest rated measure in terms of urgency was No. 64, suggesting the inclusion of topics such as participation, empowerment, cultural competence and communication training in the curricula of health care and helping professionals in postgraduate and continuing studies. The significance of these topics is supported by previous research, suggesting that frontline helping professionals have substandard practices towards Roma and should be supported in accommodating to the culture-bound and structural vulnerabilities of their patients [60, 73]. The importance of training health care professionals on how to approach such patients is also reflected in Czech policy papers [58]. Our findings are in line with previous studies explaining that such competencies can facilitate the elimination of ethnic disparities in health care [74]. Another priority measure (No. 9) contains the engagement of medical students as field health professionals within their compulsory internship. This measure could bring a partial solution to existing regional disparities [66]. Similar programmes of different length are implemented in many low and middle-income countries to ensure human resources for health in rural areas [75]. Regarding the Czech Republic, no such internship programme for medical students targeting socially excluded localities or Roma living in social exclusion has been implemented. Nevertheless, we assume it has the potential to bring the expected benefits if implemented with an emphasis on a comprehensive master plan, clearly articulated programme goals, appropriate education and training, transparent recruitment and placement, strong institutional and system support, competitive benefits and incentives, and active management of exit from the programme, as suggested in the scoping review by Antonio et al. [75]. Moreover, future professionals involved in such program would gain valuable experiences and a better understanding of the target group. The last of the top three priority measures discussed (No. 8) targeted the health-needs assessment of Roma living in social exclusion to set up interventions. The need for demand-oriented research bringing evidence-based recommendations for policy and practice in the area of Roma health has been articulated several times [19, 76]. Since the needs of the target group should be taken into account in particular [76, 77], the proposed measure is fully in line with the above-mentioned recommendations and may be conducted by independent research institutions.

Our findings contribute to the ongoing debates about health equity for Roma and bettering their position in relation to their health and access to health care. Priority measures are focused not only on overcoming barriers in access to health care for Roma living in social exclusion but also on barriers posed by the health care system itself. Recommended measures emphasize the need to increase the quality of services and cooperation of helping professionals more than improving the health literacy of Roma. It is quite a different attitude than in current national policy documents, where the individual responsibility for health is emphasized more than structural terms [58].

The main strength of this paper is its studied topic, which is important and up-to-date but has been thus far underrepresented in the available literature; based on our knowledge, it is the first study of its kind in the Czech Republic. Also, the quality of the sample can be seen as a strength, too, as it ensured a wide variety of viewpoints from diverse stakeholders from research, policy and practice engaged in the studied topic and/or responsible for some parts of it. Stakeholders proposed a set of relevant and acceptable measures, which may help improve access to health care for Roma living in social exclusion.

However, some limitations need to be mentioned as well. The methodology used might be prone to social desirability. Also, the cross-sectional design of our study limits the potential to draw causal conclusions from the findings.

## Conclusions

Participants identified six main areas that can help to improve access to health care for Roma living in social exclusion in the Czech Republic: streamlining the health care system and associated services to legitimize and take into account the needs of target group, the role of the health-promotion services and access to health care, education and awareness of the target group, increasing the local and financial accessibility of health care, strengthening the networks and capacities of health care and prevention, and education and supervision of the helping professions. Participants viewed measures related to education and supervision of helping professions as the most urgent and feasible and those related to increasing the local and financial accessibility of health care as the opposite. Two clusters were rated contradictorily. The one related to education and awareness of the target group was rated as the least urgent and the most feasible, and a second one related to strengthening the networks and capacities of health care participants was rated as very urgent but less feasible. Increasing the local and financial accessibility of health care for the target group was rated as less urgent and the least feasible. On one hand, the proposed measures mirror the most acute policy issues identified by several European and national bodies; on the other hand, the way our participants rated the proposed measures reflects current unfavourable mainstream and public discourse concerning Roma living in social exclusion. Overall, all the proposed measures are in line with European priorities and recommendations on improving access to health care for vulnerable groups, making them relevant and acceptable for implementation at the local as well as the national level.

## Supplementary Information

**Additional file 1.**

## Data Availability

The datasets used and/or analysed in the current study are available from the corresponding author on reasonable request.

## Abbreviations

CM: Concept Mapping
EU: European Union
NGOs: Non-governmental organizations
No.: Number
SD: Standard deviation
WHO: World Health Organization.
