# Supplementary material for: How to improve access to health care for Roma living in social exclusion: a concept mapping study
Source: Int J Equity Health. 2021 Feb 12;20:61. doi: 10.1186/s12939-021-01396-4 (PMC7881662; doi:10.1186/s12939-021-01396-4)
Supplement: Supplementary file 1 — Additional file 1. [file 12939_2021_1396_MOESM1_ESM.docx]

**Additional file 1**

This additional file contains a list of all measures sorted into 6 groups as specified by the participants. The numbers of 21 of them are shaded, which means that participants rated them as most urgent and feasible (belonging to the “Go-Zone”).

| **CLUSTER 1 - Streamlining the health care system and associated services to legitimize and take into account the needs of the target group** | | |
| --- | --- | --- |
| 1 | To prevent patients from terminating inpatient health care prematurely (reversal) for social and economic reasons (e.g. to provide childcare; changing the settings of the carer’s allowance such that longer-term hospitalization of the household member for whom the carer’s allowance is taken does not lead to its non-payment) | |
| 2 | To promote the collection of ethnic data and/or data on people living in social exclusion in the Czech Republic (statistics) in the area of disease and health of the population as the basis for decision-making on the national level | |
| 5 | Strategic litigation in the field of unequal treatment in health care (or litigation in the public interest) – court proceedings, whose aim is the introducing of social changes, a precedent – in this case, court proceedings in a matter of discrimination in health care based on race, ethnic origin, nationality | |
| 10 | To strengthen transport links to socially excluded localities | |
| 40 | Creation of a multidisciplinary team of field workers (social worker + medic – doctor/nurse/psychologist) that will provide care in a client’s natural environment, on the model of the Mental Health Centres that are being established as part of psychiatric care reform in the Czech Republic | |
| 41 | Creation of multidisciplinary teams for managing serious cases requiring the cooperation of experts from several areas (a social worker, school assistant or social pedagogue, medic, psychologist, etc.) | |
| 47 | To clearly define the competencies and scope of organizations focusing on health promotion and health care access for the regional balance of the network of helping organizations (networking, coordination of projects) | |
| 55 | To strengthen the level of health care and prevention in socially excluded localities through the use and training of volunteers from the community with the subsequent opportunity to later work as a “health mediator/health assistant” | |
| 3 | To create a platform of organizations that would advocate health support for people living in social exclusion, including Roma, and try to influence public policy on this sense | |
| 7 | Evaluation of all current interventions focused on health and health care access | |
| 8 | To explore the field of health needs of Roma living in social exclusion: what they need and expect from health care to set up interventions in the helping professions | |
| 51 | To ensure help in the target population with health-needs management, monitoring patient cooperation in treatment, accompanying clients to the doctor and involving the family and community in the process of health care/treatment | |
| 54 | To ensure the connection of clients from the target population with health care providers (strengthening their trust, lowering barriers) through health assistants and health mediators | |
| 63 | To link health care with the social services available in a region (inform health care professionals about the offer and competencies of social services in the given locality, so that they are capable of turning to social services as needed and invite them to cooperate with patients – e.g. a medical counsellor for doctors, a social service mobile app) | |
|  |  | |
| **CLUSTER 2 – The role of health promotion services and access to health care** | | |
| 6 | | Reducing the stigma associated with the use of medical services (mental health, reproductive health, etc.) |
| 21 | | To establish an online application for improved navigation in the health care system |
| 46 | | To enable providers of health care services to request from some providers the assigning of “health mediators/health assistants” to primary care clinics (a general practitioner for adults, paediatric general practitioner, dentist and gynaecologist) to assist in preventive examinations, education, with searching for specialists, monitoring adherence to a treatment regime, etc. |
| 48 | | To ensure the continuity of the position of “health mediators/health assistants” by introducing the position into legislation, into the catalogue of jobs and in particular by financing them from the state budget with the designation of the responsible ministry |
| 56 | | Support of the sustainability and expansion of the network of “health mediators/health assistants” to all regions (fieldwork in the area of health promotion and health care access) ideally from among members of socially excluded communities with appropriate accredited training |
| 59 | | Strengthening the health promotion agenda, including the availability of health care in social prevention services (in the work of social workers and social services workers) with appropriate accredited training |
| **61** | | To create space for the presentation of the work of “health mediators/health assistants" (field community work in the area of health promotion, including access to health care) for doctors and other medical professionals (in the form of a health conference or a demonstration of the services) |
|  | | |
| **CLUSTER 3 – Education and awareness of the target group** | | |
| **12** | | Establishment of a toll-free non-stop Infoline for providing information in the field of health and navigation of health care and/or use the existing network of such toll-free lines for this purpose and promotion |
| **18** | | To inform clients being listed and delisted from Labour Office records about the contexts of health and social insurance and to provide them support with the risk-free resolution of a situation |
| **20** | | To use social networks for raising awareness about health and health care |
| **22** | | Education targeted on health literacy in waiting rooms on television screens in the form of spots on the rights and obligations of patients, on diseases and health and health care |
| **25** | | To create and distribute printed materials on the rights and obligations of a person when at the doctor, under medical care, when to go for prevention, etc. |
| **49** | | Education of clients regarding medicines without a co-payment and alternatives when picking up medicine |
| **50** | | Education of clients on the change/loss of insurance and their obligations in the area of health care provision |
| **52** | | To ensure the education of clients on their rights, procedures for asserting their rights and filing complaints |
| **53** | | To ensure increased health literacy and motivation of the target population regarding health care and adequate use of health care |

| **CLUSTER 4 –**  **Increasing the local and financial accessibility of health care** | |
| --- | --- |
| **4** | Presentation of topics on health care accessibility in the parliamentary committee/in the Chamber of Deputies to push the agenda in the creation of measures at the government level |
| **11** | To ensure a network of pharmacies/access to medicine in regions at risk of social exclusion, where pharmacies have been shut down or are completely lacking (e.g. Jesenicko, Žulovsko) |
| **13** | To provide free contraception (basic types of tablets and bodies) for all (possibly to be means-tested) |
| **14** | To reassess and expand the basic offer of medicines with no co-payment and ensure its implementation in practice |
| **15** | Reimbursement of travel costs for travelling to see a doctor for recipients of the material needs benefit |
| **16** | To expand the possibilities and offer of drawing free benefits and contributions of health insurance companies (meaningful for the target group, e.g. credit for medicines,…) for people living in social exclusion **(**possibly to be means-tested) and information about them |
| **17** | To reduce or perhaps eliminate emergency room fees |
| **19** | To reduce supplementary fees/increase contributions for compensatory aids from insurance companies (possibly to be means-tested) |
| **23** | To introduce regular preventive paediatric and dental care in nursery schools and primary schools |
| **27** | To ensure, in cooperation with insurance companies, reserve capacity with health care providers (particularly GPs, paediatricians and dentists) for families that move frequently or have secured housing for several weeks/months, for example, so that they do not have to change doctors or commute to other cities every time (e.g. the possibility of seeing a different paediatrician for vaccinations, scheduled examinations or other non-acute needs, where the medical situation does not require an emergency room visit) |
| **33** | To negotiate with health insurance companies for strengthening the capacity of doctors (including specialists) |
| **34** | To establish a general practitioner’s surgery in socially excluded localities, but only as a temporary solution |
| **35** | To introduce mobile clinics/surgeries coming to patients in localities with poor health services coverage |
| **36** | To introduce a unified electronic system for medical records (reasons: simplification for clients with higher mobility, prevention of loss of records) |
| **38** | The legal obligation of an active role for insurance companies in providing health care to patients who cannot find a doctor (through methodological instruction of the Ministry of Health, the possibility of direct intervention by an insurance company with a given doctor and pre-negotiation of patient registration, if the doctor does not declare filled capacity) |
| **62** | A common interconnected electronic system for doctors, the labour office, social security administration and health insurance companies, where they can find information about a client, whether he/she is currently incapacitated (then a sick client does not have to personally confirm the labour office), determine the degree of disability with or without payment (then he/she does not have to personally document the decision at the labour office, health insurance company and doctors), labour office registration, the receiving of material need benefit, jointly evaluated, a parental allowance, carer’s allowance, care provider (then the health insurance company will have an overview and the client does not have to personally submit these documents and no debt arises) |

| **CLUSTER 5 – Strengthening the networks and capacities of health care and prevention** | |
| --- | --- |
| **9** | To engage medical students as field health professionals within their compulsory internship |
| **26** | To make it easier legislatively for doctors from abroad to practice in the Czech Republic |
| **28** | An incentive allowance for doctors and medical facilities (dentists, gynaecologists, psychiatrists...) for the treatment or registration of clients from socially excluded localities, paid from sources outside the health insurance company (from projects, budgets of cities and municipalities, or ministries). |
| **29** | Advantages for proactive doctors who support prevention by maintaining personal contact with the patient (sending SMS invitations to preventive visits, telephone notification of an appointment date, the active offer of prevention programmes, active interest in the overall health situation and context) |
| **30** | To simplify the conditions for obtaining attestation and making areas of study (paediatrics, dentistry, child psychiatry, child clinical psychology and others) more attractive to support the capacity of doctors |
| **31** | To re-evaluate the system of benefits for outpatient physicians in unattractive regions (increased bonuses, a housing allowance, the founding of surgery) to increase interest in their use |
| **32** | To make the study of medicine conditional on the completion of a certain number of years in the Czech Republic or the payment of study costs, so that doctors do not go abroad (or at least in cases where a scholarship has been provided) |
| **37** | To support and financially evaluate the visits of doctors to patients in households in socially excluded localities |
| **39** | To change the point system for dentists such that services paid by the insurance company are advantageous for doctors even for the care of less solvent clients without the possibility of direct payments/co-payments |
| **42** | The creation of the position of “field nurse” as a supplement to the current position of a nurse in a general practitioner’s surgery |
| **45** | Creation of the position of “health mediator in hospitals” in exposed departments (gynaecological and obstetrics, neonatology, paediatric wards), whose work would take place directly in a hospital and the work activities would meet the needs of patients and staff – mediation, communication, education, prevention, help |
| **58** | The involving of students of helping professions at universities (e.g. medical disciplines, social work, medical social care, etc.) in preventive health promotion programmes, including health care accessibility |

| **CLUSTER 6 – Education and supervision of helping professions** | |
| --- | --- |
| **24** | To create an offer of accredited courses for social workers, officials, social service providers and non-profit organizations working with the target group on the topic of prevention and health care access for Roma living in social exclusion. |
| **43** | To ensure accessible supervision in the form of lifelong learning for health professionals providing care to the target population and for “health mediators/health assistants” |
| **44** | Education of midwives so that they can work with new mothers in obstetrics wards; education of women after childbirth in the maternity hospital (care of the self and the new-born child) |
| **57** | To ensure a quality workload of training for “health mediators/ health assistants” (fieldwork in the area of health promotion, including health care access) under the sponsorship of a professional guarantor based on good practices in our country and abroad (involve organizations that are already working on it) and to ensure the possibility of exchange stays |
| **60** | To motivate Roma children to study health disciplines and to educate and support Roma health professionals (doctors, nurses, public health protection and support assistants, midwives, paramedics, orderlies) – subsidies for scholarship and mentoring programmes (securing a network of mentors with credit motivation for students) |
| **64** | To educate health care and helping professionals in postgraduate studies and continuing education (equal access and ethics, participatory and supportive – empowering approaches at work, the specifics of culturally different groups of patients and related training in communication skills of health professionals related to them) |
